# Temperature-dependent phenotypic variation of *Campylobacter jejuni *lipooligosaccharides

**DOI:** 10.1186/1471-2180-10-305

**Published:** 2010-11-30

**Authors:** Evgeny A Semchenko, Christopher J Day, Jennifer C Wilson, I Darren Grice, Anthony P Moran, Victoria Korolik

**Affiliations:** 1 Institute for Glycomics, Griffith University, Gold Coast campus, Queensland, Australia; 2 Microbiology, School of Natural Sciences, National University of Ireland, Galway, Ireland

## Abstract

**Background:**

*Campylobacter jejuni *is a major bacterial cause of food-borne enteritis, and its lipooligosaccharide (LOS) plays an initiating role in the development of the autoimmune neuropathy, Guillain-Barré syndrome, by induction of anti-neural cross-reactive antibodies through ganglioside molecular mimicry.

**Results:**

Herein we describe the existence and heterogeneity of multiple LOS forms in *C. jejuni *strains of human and chicken origin grown at 37°C and 42°C, respectively, as determined on sodium dodecyl sulphate-polyacrylamide electrophoresis gels with carbohydrate-specific silver staining and blotting with anti-ganglioside ligands, and confirmed by nuclear magnetic resonance (NMR) spectroscopy. The *C. jejuni *NCTC 11168 original isolate (11168-O) was compared to its genome-sequenced variant (11168-GS), and both were found to have a lower-M_r _LOS form, which was different in size and structure to the previously characterized higher-M_r _form bearing GM_1 _mimicry. The lower-M_r _form production was found to be dependent on the growth temperature as the production of this form increased from ~5%, observed at 37°C to ~35% at 42°C. The structure of the lower-M_r _form contained a β-D-Gal-(1→3)-β-D-GalNAc disaccharide moiety which is consistent with the termini of the GM_1_, asialo-GM_1_, GD_1_, GT_1 _and GQ_1 _gangliosides, however, it did not display GM_1 _mimicry as assessed in blotting studies but was shown in NMR to resemble asialo-GM_1_. The production of multiple LOS forms and lack of GM_1 _mimicry was not a result of phase variation in the genes tested of NCTC 11168 and was also observed in most of the human and chicken isolates of *C. jejuni *tested.

**Conclusion:**

The presence of differing amounts of LOS forms at 37 and 42°C, and the variety of forms observed in different strains, indicate that LOS form variation may play a role in an adaptive mechanism or a stress response of the bacterium during the colonization of different hosts.

## Background

*Campylobacter jejuni *is now well established as the leading cause of bacterial food-borne gastroenteritis worldwide [1,2]. Infection symptoms vary in severity and may include nausea, severe or bloody diarrhea, abdominal cramping and fever [3]. *C. jejuni *infection is usually self-limiting, but in some cases may progress to the debilitating, polyneuropathic disorders Guillain-Barré syndrome (GBS) or the oculomotor variant Miller Fisher syndrome (MFS) [4,5]. Importantly, *C. jejuni *is the commonest antecedent infection in these neuropathies and expression of carbohydrate epitopes mimicking host gangliosides is considered a prerequisite for neuropathy development since such mimicry can induce pathogenic, cross-reactive antibodies [6,7]. Gangliosides are glycosphingolipids occurring in high concentration in the peripheral nervous system, particularly in the nerve axon [8]. A humoural response against these glycolipids (*e.g. *anti-GM_1_, GM_1b_, GD_1a_, GalNAc-GD_1a _GT_1a _and GQ_1b _antibodies) plays a central role in GBS and MFS development [6,7]. Mimicry of the saccharide component of gangliosides within the outer core of *C. jejuni *lipooligosaccharides (LOS) is well documented [9,10]. Supporting a pathogenic role of *C. jejuni *in GBS, *C. jejuni *LOS-induced anti-GM_1 _ganglioside antibodies react at the nodes of Ranvier, where the axon is exposed in the nerve fibre [11], resembling the pathology observed in GBS patients, and inoculation of *C. jejuni *GM_1_-mimicking LOS has been reported to induce GBS-like symptoms in a rabbit model [12].

*C. jejuni *is capable of growth at temperatures ranging from 30 to 47°C and therefore is capable of growth at the body temperatures of human and avian hosts, 37 and 42°C, respectively [13,14]. Different temperature environments may trigger events to accommodate the colonization, commensalism, pathogenesis or dormancy of this bacterium. Over 350 genes have been reported to be differentially expressed at 37°C compared to 42°C, including the *galE *and *wlaE *genes found in the LOS biosynthesis locus [15]. Moreover, LOS is an important pathogenic factor of *C. jejuni*. Arising from this, it is possible that *C. jejuni *LOS expression is affected by temperature, whether it is by variable gene expression or at the enzymatic activity level. Although mimicry of gangliosides by *C. jejuni *LOS has been extensively studied structurally over the last two decades [9,10], it is important to note that these previous characterization studies have been performed on strains grown at 37°C.

The human isolate *C. jejuni *NCTC 11168 has been a basis for studying this bacterial species since the late 1970s. The sequencing and annotation of its genome was published by the Sanger Centre [16]. A later study revealed that the genome-sequenced strain of *C. jejuni *NCTC 11168 (11168-GS) is a poor colonizer of 1 day-old chicks and showed that this variant had an altered morphology and a different transcriptional profile compared with the original NCTC 11168 isolate (11168-O) [17]. Recurrent passaging of *C. jejuni *11168-O in laboratory conditions was considered responsible for this variation.

To date, a number of genes from the LOS biosynthesis cluster of *C. jejuni *NCTC 11168 (HS:2) have been characterized [4,18] and the structures of the lipid A and saccharide components of the LOS have been reported [19-21]. The LOS outer core mimics the oligosaccharide (OS) region of GM_1 _ganglioside [20,21] and is likely to be capable of switching from a GM_1_-like epitope to a GM_2_-like epitope as a result of phase variation [22,23].

The lack of knowledge of the structure of *C. jejuni *LOS at 42°C compared to 37°C prompted us to examine the effect of incubation temperature on the phenotypic variation of LOS, including the mimicry of gangliosides, in *C. jejuni *11168-GS and 11168-O. Variation in LOS structure was assessed by electrophoretic analysis and immunoblotting and confirmed by nuclear magnetic resonance (NMR) spectroscopy. Carbohydrate epitopes produced by both strains were assessed for ganglioside mimicry using various anti-ganglioside ligands (*i.e. *antibodies, lectins and cholera toxin) as probes. In addition, LOS structural variation at these two incubation temperatures was examined in minimally subcultured *C. jejuni *isolates from humans and chickens. Importantly, notable differences were observed in the relative production by *C. jejuni *of varying size and ganglioside mimicries at 37°C and 42°C.

## Results

### Electrophoretic analysis of *C. jejuni *LOS preparations

**Figure 1:**
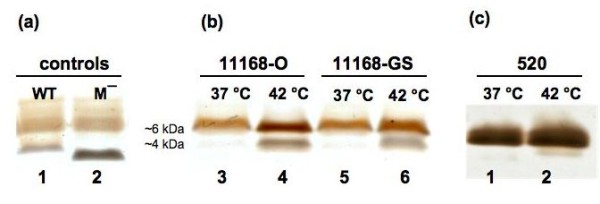
**Silver-stained SDS-PAGE gel of the LOS extracted from *C. jejuni *NCTC 11168 and 520**. (a) Controls of *M. catarrhalis *serotype A (strain 2951) LOS for relative sizing LOS. Lanes: 1, *M. catarrhalis *wild-type LOS (WT); 2, *M. catarrhalis *M¯mutant LOS lacking the terminal glucose; (b) *C. jejuni *11168-O and 11168-GS LOS extracted from bacteria grown at 37°C and 42°C. Lanes: 3, 11168-O at 37°C; 4, 11168-O at 42°C; 5, 11168-GS at 37°C; 6, 11168-GS at 42°C. (b) *C. jejuni *520 LOS extracts from bacteria grown at 37°C and 42°C. Lanes: 1, 520 at 37°C; 2, 520 at 42°C. Higher-M_r _LOS resolved at ~6 kDa and lower-M_r _LOS at ~4 kDa.

Mini-preparations of LOS isolated from *C. jejuni *11168-GS and 11168-O strains grown at 37°C and 42°C were examined using sodium dodecyl suphate-polyacrylamide gel electrophoresis (SDS-PAGE) analysis. The LOS from *C. jejuni *11168-O and 11168-GS strains resolved into two distinct forms, referred to from here on as higher-M_r _and lower-M_r _LOS (Figure 1b). Two control LOS with a known size (Figure 1a) from *M. catarrhalis *serotype A (strain 2951) were used for relative sizing of *C. jejuni *LOS. The first was wild-type LOS and resolved on the SDS-PAGE with the lower band at ~4 kDa (lane 1). The second was a LOS from a *lgt4 *mutant (2951Δ*lgt4*) of *M. catarrhalis *2951, lacking one glucose and resolved at ~3 kDa [24] (lane 2). Therefore, the difference of one hexose unit corresponded to a relative migration of ~1 kDa. Accordingly, these controls were used to compare the sizes of the *C. jejuni *LOS forms (Figure 1b and 1c). The higher-M_r _form of *C. jejuni *LOS resolved at approximately 6 kDa (and corresponds to the previously described LOS bearing GM_1 _mimicry [20-23]), whereas the lower-M_r _form, which has not been previously reported, was observed at ~4 kDa. Figure 1b shows that *C. jejuni *11168-O (lanes 3 and 4) and 11168-GS (lanes 5 and 6) have a greater amount of the 4 kDa LOS form at 42°C, than at 37°C. For both 11168-O and -GS at 42°C the amount of LOS produced appears greater than at 37°C, both in terms of quantity of the 6 kDa form and the 4 kDa form. Densitometry analysis revealed that for 11168-O at 37°C (Figure 1b, lane 3) 6.3% of the total LOS produced was the 4 kDa form and 93.7% was the 6 kDa form. In contrast, at 42°C 35.5% of total LOS produced was the 4 kDa form and 64.5% was the 6 kDa form. Similar results were observed for 11168-GS variant. These results were confirmed using purified LOS preparations from *C. jejuni *11168-O and 11168-GS, which gave identical electrophoretic profiles (data not shown) as those of the LOS mini-preparations. Also, the total amount of protein isolated from the same cell populations of *C. jejuni *11168-O and *C. jejuni *11168-GS were unaffected by the change of growth temperature (data not shown), thus allowing normalisation of cell samples prior to proteinase K digestion to produce LOS mini-preparations for comparison. In contrast to LOS, the CPS profiles from the same populations were unaffected by change of growth temperature (data not shown).

The LOS of the wild-type human isolate *C. jejuni *520 was analysed identically (Figure 1c) to determine whether the temperature-related phenomenon was unique to *C. jejuni *NCTC 11168. The LOS of strain 520 was found also to separate into the two distinct forms; the higher-M_r _and lower-M_r _LOS form. The relative LOS form profile of *C. jejuni *520 was also noted to be affected by growth temperature (Figure 1b), whereby a slightly greater amount of the lower-M_r _LOS was produced at 42°C (lane 2).

### NMR spectroscopic analysis of the higher-M_r _and lower-M_r _LOS form of *C. jejuni *111168 at 42°C

**Figure 2:**
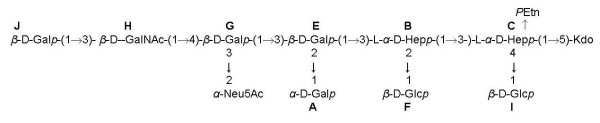
***C. jejuni *NCTC 11168 core OS structure**. Shown is the structure of the higher-M_r _LOS form [20,21], the lower-M_r _form can lack the Neu5Ac residue thereby producing an asialo-GM_1 _mimic. Abbreviations: Gal, galactose; GalNAc, *N-*acetylgalactosamine; Glc, glucose; Hep, heptose; Neu5Ac, *N-*acetylneuraminic acid Kdo, 3-deoxy-D-*manno*-oct-2-ulosonic acid; *P*Etn, phosphorylethanolamine.

**Figure 3:**
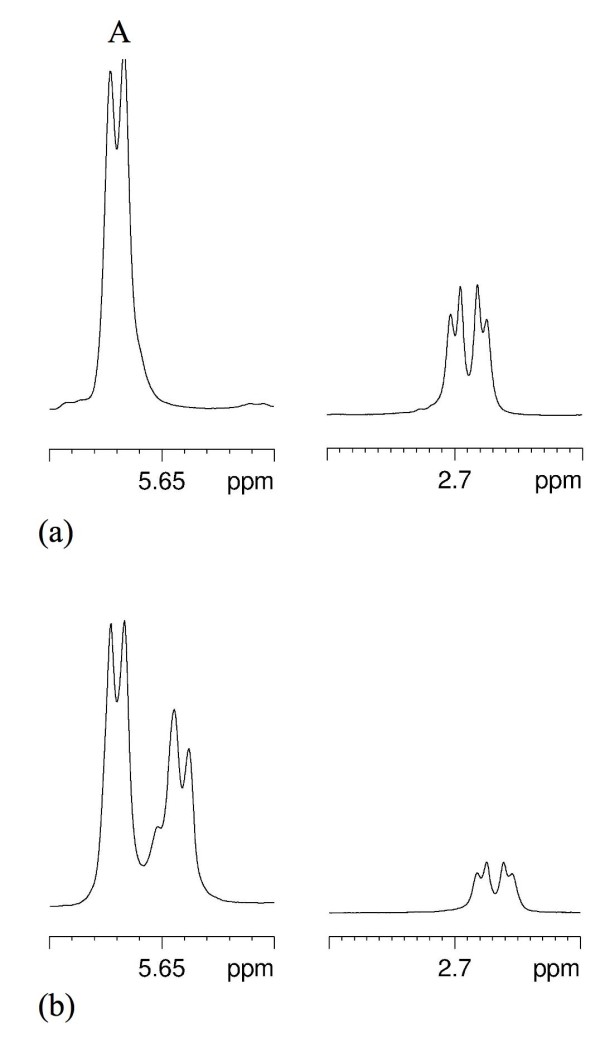
**^1^H 1D spectrum (298 K, 600 MHz) of the *C. jejuni *NCTC 11168 OS**. (a) The major fraction. (b) The minor fraction. The anomeric signal of residue **A **is shown (between 5.62 - 5.70 ppm) and the H3eq proton of α-Neu5Ac (between 2.65-2.85 ppm).

Analysis of the OS isolated from *C. jejuni *11168-O at 37°C with 1D NMR gave spectra (data not shown) consistent with the previously published structure of *C. jejuni *NCTC 11168 [20,21] (Figure 2). Given that the previous structural studies of *C. jejuni *NCTC 11168 core OS [20,21] had been performed on bacteria grown at 37°C it was of interest to investigate the differences in the core OS structure that were observed at 42°C. To this end, bacteria were grown at 42°C, the LOS extracted and purified, and the core OS acid-liberated. Examination of the ^31^P spectrum of the OS so obtained, showed a single ^31^P peak at ~0 ppm, and which was confirmed from a heteronuclear single quantum coherence (HSQC)-total correlation spectroscopy (TOCSY) spectrum to be a phosphorylethanolamine (*P*Etn) residue. Doubling up of the anomeric line of the signal attributed substitution to the →3,4,6)-L-α-D-Hep- (**C**) which is probably due to some heterogeneity in the phosphorylation of the heptose (see Figure 2). Signals consistent with α-linked *N*-acetylneuraminic acid (α-Neu5Ac, sialic acid), and *N*-acetylgalactosamine (GalNAc) were also noted. Furthermore, the anomeric region of the HSQC spectrum revealed the presence of nine anomeric signals, in addition to the α-Neu5Ac. Taken together, these spectra were consistent with the previously published structure of *C. jejuni *NCTC 11168 grown at 37°C [21] as shown in Figure 2. Nevertheless, examination of the NMR spectra of another isolated minor fraction of the core OS of 11168-O grown at 42°C revealed that there was heterogeneity in the fractions with regards to the sialylation of residue (**G**). Two separate regions of the 1D ^1^H are shown in Figure 3; a portion of the anomeric region (5.56-5.70 ppm) and the region of the spectrum where the H3eq protons of α-Neu5Ac are expected (2.65-2.85 ppm). Spectrum 3a shows the major fraction consistent with that published in [21]. In spectrum 3b, the anomeric proton found at 5.67 ppm (residue **A**) is doubled up and there is a concomitant decrease in the signal intensity of H3eq protons of Neu5Ac. The anomeric resonance of **A **is distinct from the other anomeric resonances and conveniently provides a monitor of the structure of the OS in its vicinity. It is expected that the chemical shift of the anomeric resonance of **A **would be affected by differences in the sialylation of the galactose (Gal) residue (**G**). Accordingly, in the minor fraction, which has less sialylation of residue (**G**), there is the appearance of a new anomeric signal of residue **A **at 5.64 ppm.

Collectively, the NMR data shows that there is a difference in sialylation between the higher-M_r _form of *C. jejuni *11168 LOS (~6 kDa) and the lower-M_r _form (~4 kDa); in the latter Neu5Ac can be absent, thus exhibiting asialo-GM_1 _mimicry. Sialic acid is a 9-carbon sugar and has different charge properties to hexose sugars, which accounts for the approximately 2 kDa difference in apparent mass of the two LOS forms as seen in Figure 1.

### Analysis of GM_1 _epitope mimicry in *C. jejuni *LOS using cholera toxin subunit B (CTB)

**Figure 4:**
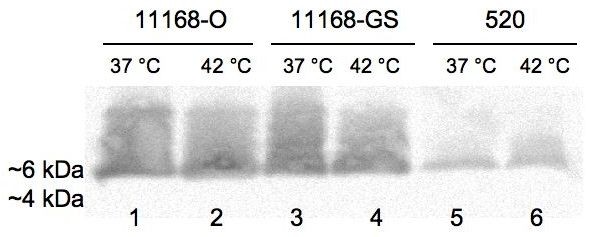
**Cholera toxin blot of the LOS extracts from *C. jejuni *11168-O, 11168-GS and 520 grown at 37°C and 42°C**. Lanes: 1, 11168-O at 37°C; 2, 11168-O at 42°C; 3, 11168-GS at 37°C; 4, 11168-GS at 42°C; 5, 520 at 37°C; 6, 520 at 42°C. A control lane without blotted material did not show reactivity (not shown). Positive binding to the higher-M_r _LOS, resolved at ~6 kDa.

*C. jejuni *11168-GS has been previously reported to mimic the structure of the GM_1 _ganglioside and hence displays strong binding to CTB [20-23,25]. Therefore, to determine whether the higher- or lower-M_r _LOS forms of *C. jejuni *11168-O and 11168-GS mimic the GM_1 _epitope, the ability of both LOS forms to bind CTB was analysed using a blotting assay. The higher-M_r _LOS of *C. jejuni *11168-O and 11168-GS isolates grown at 37°C or 42°C bound CTB strongly (Figure 4, lanes 1-4). On the other hand, the lower-M_r _LOS did not bind to CTB, indicating that it does not exhibit GM_1 _mimicry. In contrast, the higher-M_r _LOS form of *C. jejuni *strain 520 grown at 37°C or 42°C bound CTB weakly, indicating that the saccharide terminus may exhibit some ganglioside-related mimicry, though probably not GM_1_. Binding of CTB to the lower-M_r _form was not detected (Figure 4, lanes 5 and 6).

### Analysis of *C. jejuni *LOS epitope mimicry using peanut agglutinin (PNA)

**Figure 5:**
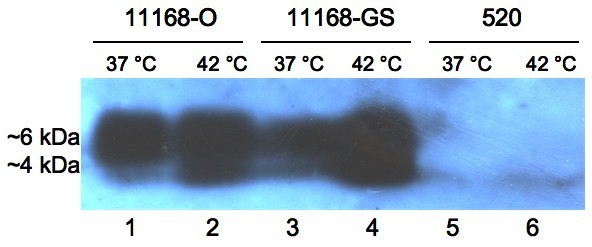
**PNA lectin blot of the LOS extracts from *C. jejuni *11168-O, 11168-GS and 520 grown at 37°C and 42°C**. Lanes: 1, 11168-O at 37°C; 2, 11168-O at 42°C; 3, 11168-GS at 37°C; 4, 11168-GS at 42°C; 5, 520 at 37°C; 6, 520 at 42°C. A control lane without blotted material did not show reactivity (not shown). Positive binding to higher-M_r _LOS resolved at ~6 kDa and lower-M_r _LOS at ~4 kDa.

To further investigate the molecular mimicry of *C. jejuni *11168 LOS forms, lectin blotting was performed using PNA which binds β-D-Gal-(1→3)-D-GalNAc and β-D-Gal(1→3)-D-Gal. The disaccharide β-D-Gal-(1→3)-D-GalNAc is present as the terminal disaccharide of GM_1 _ganglioside, but is also present in other gangliosides (*e.g. *asialo-GM1, GD_1_, GT_1 _and GQ_1 _gangliosides). PNA strongly bound both the higher-M_r _and lower-M_r _LOS forms of *C. jejuni *11168-O and 11168-GS grown at 37 and 42°C (Figure 5, lanes 1-4). Binding of the PNA to the higher-M_r _LOS is consistent with the presence of GM_1_-like mimicry and CTB binding observed above. Binding of PNA to the lower-M_r _LOS is also probably due to the occurrence of a terminal β-D-Gal-(1→3)-D-GalNAc in the truncated lower-M_r _LOS. Taking the results of CTB and PNA together suggests that the most likely structure for the lower-M_r _LOS form is an asialo-GM_1_-like structure.

In contrast, both higher-M_r _and lower-M_r _LOS of *C. jejuni *520 did not bind PNA (Figure 5; lanes 5-6) in a similar blotting procedure. This finding was consistent with the results of CTB-binding analysis of the LOS with this strain and indicated the absence of GM_1_-like mimicry, but does not exclude other ganglioside mimicry in the LOS forms of *C. jejuni *520.

### Analysis of LOS from *C. jejuni *NCTC 11168-O single colonies

**Figure 6:**
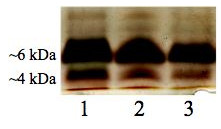
**Silver-stained SDS-PAGE gel of LOS extracted from single colonies of *C. jejuni *11168-O grown at 42°C**. Lanes: 1-3, LOS from selected individual colonies. Higher-M_r _resolved at ~6 kDa and lower-M_r _LOS observed at ~4 kDa.

To determine whether the production of multiple LOS forms occurs as a result of a phase variation, LOS mini-preparations from 30 randomly selected, single colonies of *C. jejuni *11168-O grown at 37 or 42°C were analysed. Higher- and lower-M_r _LOS forms were present within each clonal population of *C. jejuni *11168-O grown at 37 or 42°C. Figure 6 shows a representative sample of LOS profiles from single colonies grown at 42°C which showed identical profiles with ~35.5% of the total LOS produced being of 4 kDa form and ~64.5% of the 6 kDa form. LOS profiles for single *C. jejuni *11168-O colonies grown at 37°C were also identical to each other and to that shown in Figure 1b, lane 3 (data not shown). Equally strong binding of CTB to higher-M_r _LOS was observed for all the colonies tested suggesting that the phenomenon is unlikely to have been caused by phase variation. This was further confirmed by DNA sequence analysis of homopolymeric G- and A-tracts in *wlaN *and *cj1144-45c *genes as described below.

The observed GM_1 _mimicry of the LOS epitope was also confirmed by a colony lift whereby each of the single colonies of *C. jejuni *11168-O grown at 37°C was found to bind the GM_1_-binding ligand CTB (data not shown).

### Analysis of the homopolymeric tracts from the phase variable genes *wlaN *and *cj1144-45c *in *C. jejuni *NCTC 11168-O single colonies

To further examine the nature of LOS variation in *C. jejuni*, gene expression of the homopolymeric regions of two known phase variable genes, *wlaN *(responsible for addition of terminal Gal to OS [23]) and *cj1144-45c *(function unknown), located in the LOS biosynthesis locus of *C. jejuni *were analysed. Both genes were amplified from 20 randomly selected single colonies of *C. jejuni *11168-O grown at 42°C and were subsequently sequenced. Each clonal population contained an 8-residue G-tract in the *wlaN*, which allows for complete translation of the gene. The sequence of *c1144-45c *was consistently found to contain a 9-residue G-tract which interrupts the reading frame. In addition, a homopolymeric A-tract of *cj1144-45c *was also examined and no sequence variation could be detected in any of the clonal populations. As further confirmation of the lack of phase variation in the *wlaN *and *cj1144-45c *genes, the total bacterial cell population from a confluent agar plate, was subjected to similar polymerase chain reaction (PCR) analysis and sequence analysis and consistently only a single sequence for each homopolymeric tract was detected. These analyses confirmed that the growth temperature did not induce sequence variation in the lengths of the homopolymeric G-tract and A-tract in *cj1144-45c *as well as in the G-tract of *wlaN *of *C. jejuni *11168-O.

### LOS form variation in human and chicken isolates of *C. jejuni*

**Figure 7:**
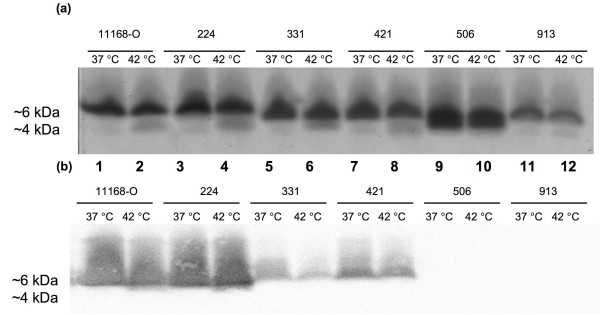
**Analysis of the LOS extracts from *C. jejuni *strains of human and chicken origin grown at 37 and 42°C**. (a) Silver-stained SDS-PAGE gel. (b) CTB blot of LOS extracts resolved as in (a). Lanes: 1, 11168-O at 37°C; 2, 11168-O at 42°C; 3: 224 at 37°C; 4, *C. jejuni *224 42°C; 5, *C. jejuni *331 37°C; 6, *C. jejuni *331 42°C; 7, *C. jejuni *421 37°C; 8, *C. jejuni *421 42°C; 9, *C. jejuni *506 37°C; 10, *C. jejuni *506 42°C; 11, *C. jejuni *913 37°C; 12, *C. jejuni *913 42°C. A control lane without blotted material did not show reactivity (not shown). Positive binding of the CTB to the higher-M_r _LOS resolved at ~6 kDa.

*C. jejuni *strains originally isolated from human patients and broiler chickens were examined to determine whether multiple LOS forms are common in *Campylobacter *strains (Table 1). Figure 7a illustrates the diversity of the LOS forms observed in extracts from a representative selection of human and chicken isolates of *C. jejuni *from those listed in Table 1. *C. jejuni *chicken isolates strains 331, 434, 506, 7-1 and RM1221 expressed both higher and lower-M_r _LOS forms whereas in strains 913, 019 and 008 only the higher-M_r _LOS form was detected (Table 1). All the human isolates were found to express both higher- and lower-M_r _LOS forms except for strain 375 where only one M_r _form (higher- M_r _form) was detected (Table 1). *C. jejuni *strains 331 (chicken), 434 (chicken), 224 (human), 421 (human) and 11168 (human) were also shown to increase the production of lower-M_r _LOS form, and a corresponding total increase in LOS production, at 42°C in contrast to 37°C (Table 1).

**Table 1:** Summary of the LOS phenotypes from different C. jejuni isolates.

Origin	*C. jejuni *strain	LOS phenotype at 37°C	LOS phenotype at 42°C
chicken	331*	H, L	H, L•
	434	H, L	H, L•
	506*	H, L	H, L
	913*	H	H
	008	H	H
	019	H	H
	7-1	H, L	H, L
	RM1221	H, L	H, L
human	224*	H, L	H, L•
	291	H, L	H, L
	351	H, L	H, L
	375	H	H
	388	H, L	H, L
	421*	H, L	H, L•
	520	H, L	H, L
	11168-GS*	H, L	H, L•
	81116	H, L	H, L

H, higher-M_r _LOS form

L, lower-M_r _LOS form

• Increase in the amount of LOS produced

*Shown in Figure 7.

A CTB blot of LOS from a representative selection of human and chicken isolates of *C. jejuni *(Figure 7b), demonstrated the variability in LOS expression in different strains with respect to ganglioside mimicry. Only the higher-M_r _LOS form was found to bind CTB in the tested strains. Furthermore, the higher-M_r _LOS of some *C. jejuni *strains (506 and 913) did not bind CTB, indicating the absence of GM_1 _ganglioside mimicry in both forms of LOS.

## Discussion

This study has shown that *C. jejuni *NCTC 11168-O and 11168-GS, as well as most randomly chosen chicken and human strains produce at least two distinct LOS forms when incubated at the core temperatures of human (37°C) and avian (42°C) hosts. This is consistent with previous observations that *C. jejuni *is capable of producing a variety of polysaccharide-related structures that are influenced by growth conditions, such as temperature [26].

Surface antigen modulation and generation of host-adapted variants are common attributes of many bacteria and enhance the pathogenicity and survivability of the microorganism, as well as the ability to evade the host immune response during the infection [27]. This variation may be achieved through several mechanisms, such as differential gene expression or enzymatic activity and specificity modulation, which can be triggered by a random and/or environmental stimuli [28]. It is possible to speculate that in the case of *C. jejuni *LOS, glycosyl transferases have the highest activity or are more stable promoting maximum functionality.

It is interesting to note that the growth temperature of *C. jejuni *NCTC 11168 was previously reported to influence the oxidative stress response [14]. In addition, approximately 20% of *C. jejuni *genes were reported to be up- or down-regulated in response to increasing the temperature from 37 to 42°C, including genes from the LOS and protein glycosylation clusters [15]. However, the change in LOS phenotype was not resolved to date.

In the present study, the phenotypic expression of the lower-M_r _LOS form appeared to be modulated by the growth temperature. This prompts speculation that the increased production of this form may be directly or indirectly caused by either specific changes in gene expression of the glycosyltransferases or glycosyl synthesis genes affecting LOS biosynthesis, thus leading to the production of varying amounts of the LOS forms. Due to the apparent loss of sialylation in the lower M_r _LOS structure it is likely that the variation of the structure is attributable to functional differences in the synthesis of the transport machinery of sialic acid under the different temperatures. We consider the most likely candidate for this difference to be the dual functioning enzyme, GalNAc transferase and CMP-Neu5Ac synthase, CgtA [18].

It is also tempting to speculate that the increased production of the lower-M_r _LOS form at 42°C might play a role in the bacterial-host interactions of *C. jejuni*. The increased production of the 4 kDa form which occurred at 42°C, the avian host body temperature, raises a possibility that this form could contribute to the commensalism by this bacterium in poultry [17]. The increase at 37°C in the proportion of the higher M_r _LOS, the portion of the LOS that is sialylated and is a GM_1 _mimic [20,21], indicates an increase in the production of an LOS structure that is thought to have a role in immune evasion and survival in mammalian hosts [29]. These hypotheses, however, will require further investigation, particularly chicken and murine infection studies.

Phase variation is the most commonly described mechanism, for antigenic variation and changes in the phenotype of the microorganism. Like *Neisseria meningitidis *and *Haemophilus influenzae*, *C. jejuni *is also known to exhibit modulation of its surface polysaccharide structures as a result of phase variation [27,30]; however, this does not appear to be the case with production of the temperature-related LOS form in *C. jejuni*. Both forms were consistently produced by all clonal populations of *C. jejuni *11168-O examined in this study suggesting that modulation of LOS forms is unlikely to be caused by phase variation. Furthermore, we have analyzed the "on-off" status of phase variable genes (*wlaN *and *cj1144-45c*) in *C. jejuni *LOS biosynthesis cluster to further demonstrate that the described variation of LOS forms is not being caused by phase variation of LOS genes. *C. jejuni *11168-O grown at 42°C was used in this experiment as it shows greater abundance of the lower-M_r _LOS form, hence increasing the chance of detecting changes in phase variable genes. Lengths of the homopolymeric G and A tracts from *wlaN *and *cj1144-45c *genes did not vary in any of 20 randomly selected colonies, suggesting that these genes are under regulatory mechanisms unaffected by growth temperature and the described variation of LOS forms is not caused by variation in the lengths of the homopolymeric tracts. Furthermore, no change in the GM_1 _mimicry of the clonal populations had been observed. It is also interesting to note that not all strains of *C. jejuni *expressed multiple LOS forms irrespective of the isolation origin, human or chicken.

No differences were observed in the production of the various LOS forms between the two variants of 11168, the genome sequenced and original isolate. The higher-M_r _form of *C. jejuni *11168 (~6 kDa) exhibited GM_1_-like mimicry and, therefore, corresponded to the previously characterized LOS [20,21,23]. Studies with CTB, a well-known binder of GM_1 _ganglisoide [25], confirmed the presence of a GM_1 _mimic in this form of NCTC 11168. Similar mimicry was also detected among the higher-M_r _LOS forms of the other isolates of humans and chickens tested, but not in the lower-M_r _form of any other strains. The weak binding of CTB to the higher-M_r _LOS variant of *C. jejuni *520 reflects that the saccharide terminus may exhibit some ganglioside-related mimic, though not GM_1 _mimicry. This is shown by the CTB binding to ganglioside-related structures not just GM_1 _and PNA did not confirm the presence of a terminal β-D-Gal-(1→3)-D-GalNAc. A CTB binding affinity study showed that the lower-M_r _form of *C. jejuni *NCTC 11168 failed to bind to the lectin. Nevertheless, the results of the present study showed that it contains a β-D-Gal-(1→3)-D-GalNAc disaccharide moiety in the core consistent with production of a truncated (because of its lower molecular mass), but related form, of the NCTC 11168 structure previously described [21], and is an asialo-GM_1_-like structure.

## Conclusion

In conclusion, this study identified the presence of a lower-M_r _LOS form produced by *C. jejuni *NCTC 11168 and other clinical and avian strains. The lower-M_r _form production was growth-temperature related as higher quantities were observed at 42°C. It is tempting to speculate that the occurrence of greater quantities of this form at avian body temperature might play a role in an adaptative mechanism to aid commensal colonization of such hosts. Alternatively, changes in the relative production of the two forms of LOS at the higher temperature could be related to a stress response. Such a phenomenon has already been seen with increased oxygen tension in the growth atmosphere of *C. jejuni *influencing the structural mimicry exhibited in the LOS of this bacterium [31]. Although an intriguing phenomenon, further investigations are required to evaluate these alternate hypotheses.

## Methods

### Bacterial strains and growth conditions

The original isolate of *C. jejuni *NCTC 11168 (11168-O) that had been characterized by Gaynor *et al. *(2004) [17], *C. jejuni *11168-GS (genome-sequenced NCTC 11168) that had been sequenced and annotated at the Sanger Centre (Hinxton, Cambridge, UK) [16], and strain 81116 were kindly supplied by D.J. Newell (Veterinary Laboratories Agency, Weybridge, UK). *C. jejuni *RM1221 has been described [32] and was kindly provided by R. E. Mandrell (United States Department of Agriculture, CA, USA.). *C. jejuni *clinical isolates 224, 291, 351, 375, 388, 421, 520, and chicken isolates 008, 019, 331, 434, 506, 913, 7-1 were obtained from the Royal Melbourne Institute of Technology (Melbourne, Vic., Australia) and Griffith University (Gold Coast, Qld., Australia) culture collections. All *C. jejuni *strains were subcultured no more than once to avoid the influence of passaging. Strains were grown on blood agar, composed of Columbia agar containing 5% (v/v) defibrinated horse blood and Skirrow's antibiotic supplement (Oxoid), under microaerobic conditions (5% O_2_, 10% CO_2 _and 85% N_2_) at 37°C for 48 h and 42°C for 24 h.

### LOS preparations

#### For gel electrophoresis

Blood agar-grown bacteria were harvested in 1 mL of sterile water, washed once in 1 mL of sterile water, and lysed by heating. Prior to lysis, samples were adjusted for numbers of bacteria using the OD_600 _measurements of bacterial suspensions. Mini-preparations of LOS were prepared by treating the whole-cell extracts with proteinase K as described previously [33]. The LOS mini-preparations from single colonies were prepared by collecting and washing cells in 40 μL of sterile water and then lysing by heating. Purified *C. jejuni *LOS was prepared by subjecting the biomass to hot phenol-water treatment using 90% (v/v) aqueous phenol at 65°C for 10 min [34]. Extracted LOS was purified by enzymatic treatment as described previously [19]. The LOS preparations were made up to 15 μg/μL in distilled water prior to gel electrophoresis.

#### For NMR analysis

*C. jejuni *11168 was grown for 24 hr as described above and bacterial biomass was harvested and washed twice using phosphate-buffered saline pH 7.4 (PBS; Sigma) and centrifugation (5000 × *g*, 4°C, 15 min). Biomass was lyophilised and 21 g and 20 g dry-cell mass was collected from cultures grown at 37°C and 42°C, respectively. Dried biomass was pretreated using pronase-E [35]. Extraction of LOS was carried out using hot-phenol water technique [34]. Water-soluble LOS was purified using RNaseA, DNase II and proteinase K (Sigma) and ultra-centrifugation, as previously described [19]. The LOS were treated with 0.1 M HCl at 100°C for 2 hours to cleave the acid-labile ketosidic linkage between the core OS and lipid A [19]. The lipid A precipitate was removed by centrifugation (5000 × *g*, 4°C, 30 mins), washed and both this and supernatant were lyophilised. The supernatant was fractionated using gel-permeation chromatography on a column of Bio-Gel P4 (1 m × 2 cm) with 0.05 M pyridinium acetate (pH 4.5) as the eluent. The resultant fractions were monitored by capillary-tube spotting on silica gel 60 TLC plates (Merck), followed by charring with 20% H_2_SO_4 _in EtOH at 150°C. The water-soluble carbohydrate-containing fractions of core OS were flash-frozen in dry-ice/acetone bath and lyophilized.

### CPS and whole-cell protein preparations

For assessing CPS production, proteinase K-treated whole cell extracts were prepared as described above. Whole-cell protein samples were prepared by incubating SDS-PAGE loading buffer with *C. jejuni *biomass at 100°C for 5 min to facilitate bacterial lysis and binding of the SDS to the denatured proteins.

### Electrophoretic analyses

Equal quantities of samples, either LOS mini-preparations or purified LOS, and CPS samples were resolved on 10% (v/v) SDS-PAGE containing urea (6 M) and tricine (0.3 mM) (Tricine-SDS-PAGE) with tricine-containing cathode buffer as previously described [36]. Stacking and separating gels contained 5.5% and 10% (v/v) acrylamide, respectively. Following the electrophoresis of LOS samples, gels were fixed and the resolved molecules were detected using the carbohydrate silver staining method [37] or CPS by Alcian Blue staining [38]. Electrophoresis was conducted at 30 V for 1 h to maximize stacking and then separated at 200 V for 30 min. Whole-cell protein samples were resolved on glycine-buffered 15% (v/v) polyacrylamide gels (Glycine-SDS-PAGE) as previously described [39]. Electrophoresis was conducted at 100 V for 1.5 h. Proteins were detected by conventional Coomassie Blue staining [19]. Densitometry image analysis was performed using the QuantityOne software package (Bio-Rad). The published *M. catarrhalis *LOS from *M. catarrhalis *wild-type (strain 2951) and the *lgt4 *LOS biosynthesis mutant [24] were used as a control for relative size determination of LOS structures due to the loss of a single hexose sugar from the known OS structure.

### NMR spectroscopy

Purified OSs were dissolved in D_2_O (CIL 99.998%) and cycled through 3 steps of lyophilization/dissolution to remove exchangeable protons. ^1^H and ^13^C NMR experiments were performed at 600 MHz and 150 MHz respectively at 298 K or 278 K in D_2_O using a Bruker Avance spectrometer. Chemical shifts are reported in ppm referenced to DSS. Spectral assignment was aided by recording of ^1^H 1D, gradient correlation spectroscopy (COSY), TOCSY, (60 and 120 ms mixing time), ^13^C attached proton test (APT), ^1^H-^13^C-HSQC and edited ^1^H-^13^C-HSQC (CH and CH_2 _correlations opposite sign), ^1^H-^13^C-HSQC-TOCSY and edited ^1^H-^13^C-HSQC-TOCSY (60 and 120 ms mixing time) (one bond C-H correlations opposite sign), and ^1^H-^13^C-HSQC-nuclear Overhauser enhancer spectroscopy (-NOESY), NOESY (400 ms) spectra. In addition, 1D selective TOCSY experiments were used to assist with the assignment process. All spectra were acquired using unmodified pulse sequences from the Bruker pulse sequence library.

### Ligand and Western blotting

In addition to chemical staining, the fractionated *C. jejuni *LOS was transferred from Tricine SDS-PAGE gels onto a Pall^® ^PVDF membrane using a semi-dry transblotter (Bio-Rad). After transfer, the membrane was reacted with horseradish peroxidase-(HRP-) conjugated CTB (3 μg mL^-1^), or with HRP-conjugated PNA (lectin from *Arachis hypogaea*) (5 μg mL^-1^), or with HRP-conjugated anti-GM_1 _ganglioside IgG (diluted 1:3000) in PBS. Membranes were developed using HRP Color Development Solution (Bio-Rad) or SuperSignal HRP Chemiluminescent Substrate (Thermo Scientific) according to the manufacturer's instructions.

### Colony lift

*C. jejuni *was grown on Columbia agar containing 2% (v/v) horse blood under microaerobic conditions as described above. The lower concentration of blood was used to reduce the background during the blotting procedure. Colonies were bound to the nitrocellulose membrane by overlaying the agar plate. The membrane was then baked for 1 h at 80°C as described elsewhere [40]. Subsequently, the membrane was blotted with HRP-CTB as described above.

### Amplification and sequencing of phase variable genes *wlaN *and *cj1144-45c*

For PCR *wlaN *G-tract forward (GATATAGCTAAAGAGTATGCTAGTAAAG) *wlaN *G-tract reverse (GGATAATATAATAAGGCATCTTCTGCC) and *cj1144-45c *G-tract forward (GGGTTGATGAAGCAAGAAATTAGTAG) *cj1144-45c *G-tract reverse (GCTAAAAACCAAGGTCCTATAACACC) primer combinations wee used. Twenty (20) reactions were inoculated with bacteria from single colonies of *C. jejuni *11168-O grown at 42°C. Amplified ~500 bp fragments were cleaned up using an Eppendrof Perfectprep Gel Cleanup kit and were sent for sequencing at the Australian Genome Research Facility (University of Queensland, St. Lucia, Brisbane, QLD, Australia).

## Abbreviations

CPS: capsular polysaccharide; CTB: cholera toxin subunit B; COSY: correlation spectroscopy; HRP: horseradish peroxidase; HSQC: heteronuclear single quantum coherence; GBS: Guillain-Barré syndrome; Gal: galactose; GalNAc: galactosamine; LOS: lipooligosaccharide; M_r_: molecular mass; MFS: Miller Fisher syndrome; Neu5Ac sialic acid: (*N*-acetylneuraminic acid); NMR: nuclear magnetic resonance; OS: oligosaccharide; PBS: phosphate-buffered saline; PCR: polymerase chain reaction; *P*Etn: phosphorylethanolamine; PNA: peanut agglutinin; SDS-PAGE: sodium dodecyl suphate-polyacrylamide gel electrophoresis; TOCSY: total correlation spectroscopy.

## Authors' contributions

EAS carried out all of the electrophoretic and blotting experiments and drafted the initial manuscript. CJD aided with experimental work and participated in the design and coordination of the study and helped to draft the manuscript. IDG and JCW provided resources, aided in determination of the LOS structures with APM and helped draft the manuscript. APM and VK conceived this study, participated in its design, and the coordination and writing of the manuscript. All authors read and approved the final manuscript.
